# Retraction of The CO/HO system reverses inhibition of mitochondrial biogenesis and prevents murine doxorubicin cardiomyopathy

**DOI:** 10.1172/JCI211052

**Published:** 2026-08-17

**Authors:** Hagir B. Suliman, Martha Sue Carraway, Abdelwahid S. Ali, Chrystal M. Reynolds, Karen E. Welty-Wolf, Claude A. Piantadosi

Original citation: *J Clin Invest*. 2007;117(12):3730–3741. https://doi.org/10.1172/JCI32967

Citation for this retraction: *J Clin Invest*. 2026;136(16):e211052. https://doi.org/10.1172/JCI211052

The Editors became aware of concerns with data in Figures 2A, 5A, 5B, 5H, and 7A, including the use of duplicated images to represent distinct samples. After reviewing the matter, Duke University was unable to locate the original source data and informed the Journal that they could not support the veracity of the data in question. As we are unable to confirm the integrity of these figure panels, the Editors decided to retract the article.

The authors did not respond to contact from the journal regarding the retraction or could not be reached.

## Version 1 08/17/2026

Electronic publication

